# Retrospective analysis of main and interaction effects in genetic association studies of human complex traits

**DOI:** 10.1186/1471-2156-8-70

**Published:** 2007-10-16

**Authors:** Qihua Tan, Lene Christiansen, Charlotte Brasch-Andersen, Jing Hua Zhao, Shuxia Li, Torben A Kruse, Kaare Christensen

**Affiliations:** 1 Epidemiology, Institute of Public Health, University of Southern Denmark, Denmark; 2 Department of Biochemistry, Pharmacology and Genetics, Odense University Hospital, Denmark; 3 Clinical Pharmacology, Institute of Public Health, University of Southern Denmark, Denmark; 4 MRC Epidemiology Unit, The Strangeways Research Laboratory, Worts Causeway, Cambridge CB1 8RN, UK

## Abstract

**Background:**

The etiology of multifactorial human diseases involves complex interactions between numerous environmental factors and alleles of many genes. Efficient statistical tools are demanded in identifying the genetic and environmental variants that affect the risk of disease development. This paper introduces a retrospective polytomous logistic regression model to measure both the main and interaction effects in genetic association studies of human discrete and continuous complex traits. In this model, combinations of genotypes at two interacting loci or of environmental exposure and genotypes at one locus are treated as nominal outcomes of which the proportions are modeled as a function of the disease trait assigning both main and interaction effects and with no assumption of normality in the trait distribution. Performance of our method in detecting interaction effect is compared with that of the case-only model.

**Results:**

Results from our simulation study indicate that our retrospective model exhibits high power in capturing even relatively small effect with reasonable sample sizes. Application of our method to data from an association study on the catalase -262C/T promoter polymorphism and aging phenotypes detected significant main and interaction effects for age-group and allele T on individual's cognitive functioning and produced consistent results in estimating the interaction effect as compared with the popular case-only model.

**Conclusion:**

The retrospective polytomous logistic regression model can be used as a convenient tool for assessing both main and interaction effects in genetic association studies of human multifactorial diseases involving genetic and non-genetic factors as well as categorical or continuous traits.

## Background

The changing disease pattern has brought complex diseases as one of the significant challenges for the 21^st ^century medicine. As the etiology of complex diseases involves both multiple genetic and environmental factors combined with their interactions, statistical methods for efficiently measuring the main and interaction effects are demanded. In the literature of genetic epidemiology, the linkage disequilibrium (LD) based genetic association study, advantaged by the recent development of high-throughput SNP genotyping technology, has been the workhorse and holds the promise of mapping out susceptibility genes to complex diseases [1]. The case-control design, a retrospective design by nature, has been popular in establishing the genetic associations in single locus and haplotype analyses [2] as well as in assessing gene-environment interactions [3,4]. Recently, this approach has been extended to handle both dichotomous and continuous traits by introducing the retrospective logistic regression model [5] that treats alleles or genotypes as dependent variables. For example, the idea has been used by Waldman et al. [6] to model the probability of allele transmission as a function of offspring's trait value in family-based transmission disequilibrium test (TDT). The same idea has been used for single locus analysis in both unmatched and matched case-control studies [7] and for haplotype analysis [8].

Another contribution to genetic epidemiology by the retrospective case-control design is the introduction of nontraditional case-only design [9] for assessing gene-environment and gene-gene interactions. Measuring the interaction effects in complex disease study is important because many of the susceptibility genes act through modification of disease risk associated with other genes or environmental factors. Unfortunately, application of the case-only method is restricted to dichotomous or binary traits. Otherwise one needs to set up a cut-off on a continuous trait to define cases [10]. Although other statistical models for measuring interactions [11-13] exist, Glaser et al. [14] recently reported that different methods can give different results for the same data due to underlying assumptions.

In this paper, we introduce a retrospective polytomous logistic regression model to measure both the main and the interaction effects in genetic association studies of human complex traits which can be discrete or continuous. In this model, combinations of genotypes at two interacting loci or of environmental exposure and genotypes at one locus are treated as nominal outcomes of which the proportions are modeled as a function of the disease trait assigning both main and interaction effects. The performance of our method in detecting interaction effect is compared with that of the case-only model. A limited simulation study is performed to assess the power and type I error rate for given parameters settings. Application of our method is exemplified using data from our association study on catalase -262C/T promoter polymorphism and aging phenotypes [15] to look for both main and interaction effects of genetic variation and aging in affecting cognitive function in the Danish population.

## Methods

Suppose we are interested in assessing the main and interaction effects of one genetic variant G (allele or genotype, G = 1 for carriers and 0 for non-carriers) and environmental exposure E (E = 0 for non-exposed and 1 for exposed). The combination of G and E leads to four nominal categories. The purpose of our retrospective polytomous logistic regression model is to model the proportion of each of the categories, *p*, as a function of the disease trait by treating the proportions as responses for given trait value *x*, i.e. we model

*Logit*[*p*(*G *= *I*, *E *= *J*|*x*)] = *a*_*G*_*I *+ *a*_*E*_*J *+ (*b*_*G*_*I *+ *b*_*E*_*J *+ *b*_*G×E*_*IJ*)*x*   *I*, *J *= 0,1     (1)

where *a *and *b *are the intercept and slope parameters assigned to G and E respectively. In (1), the association of G and E with trait *x *are measured by the slope parameters with *b*_*G *_and *b*_*E *_for the main effects from G and E and *b*_*G*×*E *_for their interaction effect. Rewriting (1) as the conditional probability of each outcome category given the trait value *x*, we have

p(G=I,E=J|x)=exp⁡[aGI+aEJ+(bGI+bEJ+bGxEIJ)x]∑I'∑J'exp⁡[aGI'+aEJ'+(bGI'+bEJ'+bGxEI'J')x]I,J,I',J'=0,1     (2)
 MathType@MTEF@5@5@+=feaafiart1ev1aaatCvAUfKttLearuWrP9MDH5MBPbIqV92AaeXatLxBI9gBaebbnrfifHhDYfgasaacH8akY=wiFfYdH8Gipec8Eeeu0xXdbba9frFj0=OqFfea0dXdd9vqai=hGuQ8kuc9pgc9s8qqaq=dirpe0xb9q8qiLsFr0=vr0=vr0dc8meaabaqaciaacaGaaeqabaqabeGadaaakeaafaqabeqacaaabaGaemiCaaNaeiikaGIaem4raCKaeyypa0JaemysaKKaeiilaWIaemyrauKaeyypa0JaemOsaOKaeiiFaWNaemiEaGNaeiykaKIaeyypa0ZaaSaaaeaacyGGLbqzcqGG4baEcqGGWbaCcqGGBbWwcqWGHbqydaWgaaWcbaGaem4raCeabeaakiabdMeajjabgUcaRiabdggaHnaaBaaaleaacqWGfbqraeqaaOGaemOsaOKaey4kaSIaeiikaGIaemOyai2aaSbaaSqaaiabdEeahbqabaGccqWGjbqscqGHRaWkcqWGIbGydaWgaaWcbaGaemyraueabeaakiabdQeakjabgUcaRiabdkgaInaaBaaaleaacqWGhbWrcqWG4baEcqWGfbqraeqaaOGaemysaKKaemOsaOKaeiykaKIaemiEaGNaeiyxa0fabaWaaabuaeaadaaeqbqaaiGbcwgaLjabcIha4jabcchaWjabcUfaBjabdggaHnaaBaaaleaacqWGhbWraeqaaOGaemysaKKaei4jaCIaey4kaSIaemyyae2aaSbaaSqaaiabdweafbqabaGccqWGkbGscqGGNaWjcqGHRaWkcqGGOaakcqWGIbGydaWgaaWcbaGaem4raCeabeaakiabdMeajjabcEcaNiabgUcaRiabdkgaInaaBaaaleaacqWGfbqraeqaaOGaemOsaOKaei4jaCIaey4kaSIaemOyai2aaSbaaSqaaiabdEeahjabdIha4jabdweafbqabaGccqWGjbqscqGGNaWjcqWGkbGscqGGNaWjcqGGPaqkcqWG4baEcqGGDbqxaSqaaiabdQeakjabcEcaNaqab0GaeyyeIuoaaSqaaiabdMeajjabcEcaNaqab0GaeyyeIuoaaaaakeaacqWGjbqscqGGSaalcqWGkbGscqGGSaalcqWGjbqscqGGNaWjcqGGSaalcqWGkbGscqGGNaWjcqGH9aqpcqaIWaamcqGGSaalcqaIXaqmaaaaaa@9C0B@

When *I *= *J *= 0, the numerator of (2) becomes 1 so that we have

p(G=0,E=0|x)=1∑I'∑J'exp⁡[aGI'+aEJ'+(bGI'+bEJ'+bGxEI'J')x]I',J'=0,1     (3)
 MathType@MTEF@5@5@+=feaafiart1ev1aaatCvAUfKttLearuWrP9MDH5MBPbIqV92AaeXatLxBI9gBaebbnrfifHhDYfgasaacH8akY=wiFfYdH8Gipec8Eeeu0xXdbba9frFj0=OqFfea0dXdd9vqai=hGuQ8kuc9pgc9s8qqaq=dirpe0xb9q8qiLsFr0=vr0=vr0dc8meaabaqaciaacaGaaeqabaqabeGadaaakeaafaqabeqacaaabaGaemiCaaNaeiikaGIaem4raCKaeyypa0JaeGimaaJaeiilaWIaemyrauKaeyypa0JaeGimaaJaeiiFaWNaemiEaGNaeiykaKIaeyypa0ZaaSaaaeaacqaIXaqmaeaadaaeqbqaamaaqafabaGagiyzauMaeiiEaGNaeiiCaaNaei4waSLaemyyae2aaSbaaSqaaiabdEeahbqabaGccqWGjbqscqGGNaWjcqGHRaWkcqWGHbqydaWgaaWcbaGaemyraueabeaakiabdQeakjabcEcaNiabgUcaRiabcIcaOiabdkgaInaaBaaaleaacqWGhbWraeqaaOGaemysaKKaei4jaCIaey4kaSIaemOyai2aaSbaaSqaaiabdweafbqabaGccqWGkbGscqGGNaWjcqGHRaWkcqWGIbGydaWgaaWcbaGaem4raCKaemiEaGNaemyraueabeaakiabdMeajjabcEcaNiabdQeakjabcEcaNiabcMcaPiabdIha4jabc2faDbWcbaGaemOsaOKaei4jaCcabeqdcqGHris5aaWcbaGaemysaKKaei4jaCcabeqdcqGHris5aaaaaOqaaiabdMeajjabcEcaNiabcYcaSiabdQeakjabcEcaNiabg2da9iabicdaWiabcYcaSiabigdaXaaaaaa@7516@

Since (3) is for the group of individuals who are neither carriers of the genetic variant nor exposed, it can serve as the reference or baseline. With that, we are able to derive the relative risks (*RR*) for the main and interaction effects at a given trait value *x *and then define the relative risk ratios (*RRR*) for comparing *RR *at two given trait values *x*_1 _and *x*_2_. To obtain RR for the main genetic effect, we set *I *= 1 and *J *= 0 so that (2) becomes

p(G=1,E=0|x)=exp⁡(aG+bGx)∑I'∑J'exp⁡[aGI'+aEJ'+(bGI'+bEJ'+bGxEI'J')x]I',J'=0,1     (4)
 MathType@MTEF@5@5@+=feaafiart1ev1aaatCvAUfKttLearuWrP9MDH5MBPbIqV92AaeXatLxBI9gBaebbnrfifHhDYfgasaacH8akY=wiFfYdH8Gipec8Eeeu0xXdbba9frFj0=OqFfea0dXdd9vqai=hGuQ8kuc9pgc9s8qqaq=dirpe0xb9q8qiLsFr0=vr0=vr0dc8meaabaqaciaacaGaaeqabaqabeGadaaakeaafaqabeqacaaabaGaemiCaaNaeiikaGIaem4raCKaeyypa0JaeGymaeJaeiilaWIaemyrauKaeyypa0JaeGimaaJaeiiFaWNaemiEaGNaeiykaKIaeyypa0ZaaSaaaeaacyGGLbqzcqGG4baEcqGGWbaCcqGGOaakcqWGHbqydaWgaaWcbaGaem4raCeabeaakiabgUcaRiabdkgaInaaBaaaleaacqWGhbWraeqaaOGaemiEaGNaeiykaKcabaWaaabuaeaadaaeqbqaaiGbcwgaLjabcIha4jabcchaWjabcUfaBjabdggaHnaaBaaaleaacqWGhbWraeqaaOGaemysaKKaei4jaCIaey4kaSIaemyyae2aaSbaaSqaaiabdweafbqabaGccqWGkbGscqGGNaWjcqGHRaWkcqGGOaakcqWGIbGydaWgaaWcbaGaem4raCeabeaakiabdMeajjabcEcaNiabgUcaRiabdkgaInaaBaaaleaacqWGfbqraeqaaOGaemOsaOKaei4jaCIaey4kaSIaemOyai2aaSbaaSqaaiabdEeahjabdIha4jabdweafbqabaGccqWGjbqscqGGNaWjcqWGkbGscqGGNaWjcqGGPaqkcqWG4baEcqGGDbqxaSqaaiabdQeakjabcEcaNaqab0GaeyyeIuoaaSqaaiabdMeajjabcEcaNaqab0GaeyyeIuoaaaaakeaacqWGjbqscqGGNaWjcqGGSaalcqWGkbGscqGGNaWjcqGH9aqpcqaIWaamcqGGSaalcqaIXaqmaaaaaa@819B@

The *RR *for the main genetic effect is then calculated as

RRG(x)=p(G=1,E=0|x)p(G=0,E=0|x)=exp⁡(aG+bGx)     (5)
 MathType@MTEF@5@5@+=feaafiart1ev1aaatCvAUfKttLearuWrP9MDH5MBPbIqV92AaeXatLxBI9gBaebbnrfifHhDYfgasaacH8akY=wiFfYdH8Gipec8Eeeu0xXdbba9frFj0=OqFfea0dXdd9vqai=hGuQ8kuc9pgc9s8qqaq=dirpe0xb9q8qiLsFr0=vr0=vr0dc8meaabaqaciaacaGaaeqabaqabeGadaaakeaacqWGsbGucqWGsbGudaWgaaWcbaGaem4raCeabeaakiabcIcaOiabdIha4jabcMcaPiabg2da9maalaaabaGaemiCaaNaeiikaGIaem4raCKaeyypa0JaeGymaeJaeiilaWIaemyrauKaeyypa0JaeGimaaJaeiiFaWNaemiEaGNaeiykaKcabaGaemiCaaNaeiikaGIaem4raCKaeyypa0JaeGimaaJaeiilaWIaemyrauKaeyypa0JaeGimaaJaeiiFaWNaemiEaGNaeiykaKcaaiabg2da9iGbcwgaLjabcIha4jabcchaWjabcIcaOiabdggaHnaaBaaaleaacqWGhbWraeqaaOGaey4kaSIaemOyai2aaSbaaSqaaiabdEeahbqabaGccqWG4baEcqGGPaqkaaa@5D1B@

Based on (5), we can calculate *RRR *for comparing *RR*s at two given trait values *x*_1 _and *x*_2 _as

RRRG=RRG(x2)RRG(x1)=exp⁡(aG+bGx2)exp⁡(aG+bGx1)=exp⁡[bG(x2−x1)]=exp⁡(kbG)     (6)
 MathType@MTEF@5@5@+=feaafiart1ev1aaatCvAUfKttLearuWrP9MDH5MBPbIqV92AaeXatLxBI9gBaebbnrfifHhDYfgasaacH8akY=wiFfYdH8Gipec8Eeeu0xXdbba9frFj0=OqFfea0dXdd9vqai=hGuQ8kuc9pgc9s8qqaq=dirpe0xb9q8qiLsFr0=vr0=vr0dc8meaabaqaciaacaGaaeqabaqabeGadaaakeaacqWGsbGucqWGsbGucqWGsbGudaWgaaWcbaGaem4raCeabeaakiabg2da9maalaaabaGaemOuaiLaemOuai1aaSbaaSqaaiabdEeahbqabaGccqGGOaakcqWG4baEdaWgaaWcbaGaeGOmaidabeaakiabcMcaPaqaaiabdkfasjabdkfasnaaBaaaleaacqWGhbWraeqaaOGaeiikaGIaemiEaG3aaSbaaSqaaiabigdaXaqabaGccqGGPaqkaaGaeyypa0ZaaSaaaeaacyGGLbqzcqGG4baEcqGGWbaCcqGGOaakcqWGHbqydaWgaaWcbaGaem4raCeabeaakiabgUcaRiabdkgaInaaBaaaleaacqWGhbWraeqaaOGaemiEaG3aaSbaaSqaaiabikdaYaqabaGccqGGPaqkaeaacyGGLbqzcqGG4baEcqGGWbaCcqGGOaakcqWGHbqydaWgaaWcbaGaem4raCeabeaakiabgUcaRiabdkgaInaaBaaaleaacqWGhbWraeqaaOGaemiEaG3aaSbaaSqaaiabigdaXaqabaGccqGGPaqkaaGaeyypa0JagiyzauMaeiiEaGNaeiiCaaNaei4waSLaemOyai2aaSbaaSqaaiabdEeahbqabaGccqGGOaakcqWG4baEdaWgaaWcbaGaeGOmaidabeaakiabgkHiTiabdIha4naaBaaaleaacqaIXaqmaeqaaOGaeiykaKIaeiyxa0Laeyypa0JagiyzauMaeiiEaGNaeiiCaaNaeiikaGIaem4AaSMaemOyai2aaSbaaSqaaiabdEeahbqabaGccqGGPaqkaaa@7DEA@

Note that, when *k *= *1 *such as in a case-control study, we have *RRR*_*G *_= exp(*b*_*G*_). In the same manner, we obtain *RRR*_*E *_= exp(*kb*_*E*_).

In order to estimate the risk of interaction effect, we set *I *= *J *= 1 so that (2) becomes

p(G=1,E=1|x)=exp⁡[aG+aE+(bG+bE+bGxE)x]∑I'∑J'exp⁡[aGI'+aEJ'+(bGI'+bEJ'+bGxEI'J')x]I',J'=0,1     (7)
 MathType@MTEF@5@5@+=feaafiart1ev1aaatCvAUfKttLearuWrP9MDH5MBPbIqV92AaeXatLxBI9gBaebbnrfifHhDYfgasaacH8akY=wiFfYdH8Gipec8Eeeu0xXdbba9frFj0=OqFfea0dXdd9vqai=hGuQ8kuc9pgc9s8qqaq=dirpe0xb9q8qiLsFr0=vr0=vr0dc8meaabaqaciaacaGaaeqabaqabeGadaaakeaafaqabeqacaaabaGaemiCaaNaeiikaGIaem4raCKaeyypa0JaeGymaeJaeiilaWIaemyrauKaeyypa0JaeGymaeJaeiiFaWNaemiEaGNaeiykaKIaeyypa0ZaaSaaaeaacyGGLbqzcqGG4baEcqGGWbaCcqGGBbWwcqWGHbqydaWgaaWcbaGaem4raCeabeaakiabgUcaRiabdggaHnaaBaaaleaacqWGfbqraeqaaOGaey4kaSIaeiikaGIaemOyai2aaSbaaSqaaiabdEeahbqabaGccqGHRaWkcqWGIbGydaWgaaWcbaGaemyraueabeaakiabgUcaRiabdkgaInaaBaaaleaacqWGhbWrcqWG4baEcqWGfbqraeqaaOGaeiykaKIaemiEaGNaeiyxa0fabaWaaabuaeaadaaeqbqaaiGbcwgaLjabcIha4jabcchaWjabcUfaBjabdggaHnaaBaaaleaacqWGhbWraeqaaOGaemysaKKaei4jaCIaey4kaSIaemyyae2aaSbaaSqaaiabdweafbqabaGccqWGkbGscqGGNaWjcqGHRaWkcqGGOaakcqWGIbGydaWgaaWcbaGaem4raCeabeaakiabdMeajjabcEcaNiabgUcaRiabdkgaInaaBaaaleaacqWGfbqraeqaaOGaemOsaOKaei4jaCIaey4kaSIaemOyai2aaSbaaSqaaiabdEeahjabdIha4jabdweafbqabaGccqWGjbqscqGGNaWjcqWGkbGscqGGNaWjcqGGPaqkcqWG4baEcqGGDbqxaSqaaiabdQeakjabcEcaNaqab0GaeyyeIuoaaSqaaiabdMeajjabcEcaNaqab0GaeyyeIuoaaaaakeaacqWGjbqscqGGNaWjcqGGSaalcqWGkbGscqGGNaWjcqGH9aqpcqaIWaamcqGGSaalcqaIXaqmaaaaaa@9113@

The RR for comparing exposed carriers to unexposed non-carriers at *x *is

RR(x)=p(G=1,E=1|x)p(G=0,E=0|x)=exp⁡[aG+aE+(bG+bE+bGxE)x]     (8)
 MathType@MTEF@5@5@+=feaafiart1ev1aaatCvAUfKttLearuWrP9MDH5MBPbIqV92AaeXatLxBI9gBaebbnrfifHhDYfgasaacH8akY=wiFfYdH8Gipec8Eeeu0xXdbba9frFj0=OqFfea0dXdd9vqai=hGuQ8kuc9pgc9s8qqaq=dirpe0xb9q8qiLsFr0=vr0=vr0dc8meaabaqaciaacaGaaeqabaqabeGadaaakeaacqWGsbGucqWGsbGucqGGOaakcqWG4baEcqGGPaqkcqGH9aqpdaWcaaqaaiabdchaWjabcIcaOiabdEeahjabg2da9iabigdaXiabcYcaSiabdweafjabg2da9iabigdaXiabcYha8jabdIha4jabcMcaPaqaaiabdchaWjabcIcaOiabdEeahjabg2da9iabicdaWiabcYcaSiabdweafjabg2da9iabicdaWiabcYha8jabdIha4jabcMcaPaaacqGH9aqpcyGGLbqzcqGG4baEcqGGWbaCcqGGBbWwcqWGHbqydaWgaaWcbaGaem4raCeabeaakiabgUcaRiabdggaHnaaBaaaleaacqWGfbqraeqaaOGaey4kaSIaeiikaGIaemOyai2aaSbaaSqaaiabdEeahbqabaGccqGHRaWkcqWGIbGydaWgaaWcbaGaemyraueabeaakiabgUcaRiabdkgaInaaBaaaleaacqWGhbWrcqWG4baEcqWGfbqraeqaaOGaeiykaKIaemiEaGNaeiyxa0faaa@6B46@

Likewise, the *RRR *for *k *= *x*_2 _- *x*_1 _is

RRR=RR(x2)RR(x1)=exp⁡[k(bG+bE+bGxE)]=exp⁡(kbG)exp⁡(kbE)exp⁡(kbGxE)=RRRGRRREexp⁡(kbGxE)     (9)
 MathType@MTEF@5@5@+=feaafiart1ev1aaatCvAUfKttLearuWrP9MDH5MBPbIqV92AaeXatLxBI9gBaebbnrfifHhDYfgasaacH8akY=wiFfYdH8Gipec8Eeeu0xXdbba9frFj0=OqFfea0dXdd9vqai=hGuQ8kuc9pgc9s8qqaq=dirpe0xb9q8qiLsFr0=vr0=vr0dc8meaabaqaciaacaGaaeqabaqabeGadaaakeaafaqaaeGadaaabaGaemOuaiLaemOuaiLaemOuaiLaeyypa0ZaaSaaaeaacqWGsbGucqWGsbGucqGGOaakcqWG4baEdaWgaaWcbaGaeGOmaidabeaakiabcMcaPaqaaiabdkfasjabdkfasjabcIcaOiabdIha4naaBaaaleaacqaIXaqmaeqaaOGaeiykaKcaaiabg2da9iGbcwgaLjabcIha4jabcchaWjabcUfaBjabdUgaRjabcIcaOiabdkgaInaaBaaaleaacqWGhbWraeqaaOGaey4kaSIaemOyai2aaSbaaSqaaiabdweafbqabaGccqGHRaWkcqWGIbGydaWgaaWcbaGaem4raCKaemiEaGNaemyraueabeaakiabcMcaPiabc2faDbqaaiabg2da9aqaaiGbcwgaLjabcIha4jabcchaWjabcIcaOiabdUgaRjabdkgaInaaBaaaleaacqWGhbWraeqaaOGaeiykaKIagiyzauMaeiiEaGNaeiiCaaNaeiikaGIaem4AaSMaemOyai2aaSbaaSqaaiabdweafbqabaGccqGGPaqkcyGGLbqzcqGG4baEcqGGWbaCcqGGOaakcqWGRbWAcqWGIbGydaWgaaWcbaGaem4raCKaemiEaGNaemyraueabeaakiabcMcaPaqaaaqaaiabg2da9aqaaiabdkfasjabdkfasjabdkfasnaaBaaaleaacqWGhbWraeqaaOGaemOuaiLaemOuaiLaemOuai1aaSbaaSqaaiabdweafbqabaGccyGGLbqzcqGG4baEcqGGWbaCcqGGOaakcqWGRbWAcqWGIbGydaWgaaWcbaGaem4raCKaemiEaGNaemyraueabeaakiabcMcaPaaaaaa@8DD8@

From (9) we obtain *RRR*_*G*×*E *_as the departure from the multiplicative effects of *RRR*_*G*_*RRR*_*E*_, i.e.*RRR*_*G*×*E *_= exp(*kb*_*G*×*E*_).

In order to estimate the parameters, we construct the following likelihood function [16]

L(aG,aE,bG,bE,bGxE)=∏n∏I,JπIJ(xn)I(Gn=I&En=J)I,J=0,1     (10)
 MathType@MTEF@5@5@+=feaafiart1ev1aaatCvAUfKttLearuWrP9MDH5MBPbIqV92AaeXatLxBI9gBaebbnrfifHhDYfgasaacH8akY=wiFfYdH8Gipec8Eeeu0xXdbba9frFj0=OqFfea0dXdd9vqai=hGuQ8kuc9pgc9s8qqaq=dirpe0xb9q8qiLsFr0=vr0=vr0dc8meaabaqaciaacaGaaeqabaqabeGadaaakeaafaqabeqacaaabaGaemitaWKaeiikaGIaemyyae2aaSbaaSqaaiabdEeahbqabaGccqGGSaalcqWGHbqydaWgaaWcbaGaemyraueabeaakiabcYcaSiabdkgaInaaBaaaleaacqWGhbWraeqaaOGaeiilaWIaemOyai2aaSbaaSqaaiabdweafbqabaGccqGGSaalcqWGIbGydaWgaaWcbaGaem4raCKaemiEaGNaemyraueabeaakiabcMcaPiabg2da9maarafabaWaaebuaeaaiiGacqWFapaCdaWgaaWcbaGaemysaKKaemOsaOeabeaakiabcIcaOiabdIha4naaBaaaleaacqWGUbGBaeqaaOGaeiykaKYaaWbaaSqabeaacqWGjbqscqGGOaakcqWGhbWrdaWgaaadbaGaemOBa4gabeaaliabg2da9iabdMeajjabcAcaMiabdweafnaaBaaameaacqWGUbGBaeqaaSGaeyypa0JaemOsaOKaeiykaKcaaaqaaiabdMeajjabcYcaSiabdQeakbqab0Gaey4dIunaaSqaaiabd6gaUbqab0Gaey4dIunaaOqaaiabdMeajjabcYcaSiabdQeakjabg2da9iabicdaWiabcYcaSiabigdaXaaaaaa@697E@

Here, *n *denotes individual observations from 1 to *N*, *π*_*IJ*_(*x*_*n*_) = *p*(*G *= *I*, *E *= *J*|*x*_*n*_), *I*(·) is an indicator function. Since our interest is only in the slope parameters, the two intercept parameters are just nuisance parameters. To obtain an overall significance of both main and interaction effects, we use the log-likelihood ratio test with *df *= 3,

*LRT *= -2[ln *L*(*a*_*G*_,*a*_*E*_) - ln *L*(*a*_*G*_,*a*_*E*_,*b*_*G*_,*b*_*E*_,*b*_*G*×*E*_)]     (11)

where *L*(*a*_*G*_,*a*_*E*_) is the likelihood of the intercept only model. Statistical test on single slope parameters can be done likewise or by introducing the Wald statistic [16].

### Simulation

In order to examine the performance of our method, we perform a limited computer simulation study to assess the power and type I error rate (*α*) when given different parameter settings. The data were simulated using a linear model, i.e. for individual *i*, we have *y*_*i *_= *β*_0 _+ *β*_1_*G *+ *β*_2_*E *+ *β*_3_G*E + *e*_*i*_. Here *y*_*i *_is a continuous phenotype value for individual *i*. G and E are the indictors for the genetic (1 for carriers and 0 for non-carriers, we set frequency of carriers to 0.2) and environmental (1 for exposed and 0 for non-exposed, exposure rate set to 0.3) variants. *β*_0 _is the intercept which we set to 0.1. *β*_1_, *β*_2 _and *β*_3 _are the slope parameters for the genetic, environmental and their interaction effects respectively. For simplicity, we assume that there is no main effect in the model, but there is an interaction effect that lowers the phenotype value for carriers for the genetic variant and who are exposed to the environment. The power for capturing the interaction effect is estimated as the frequency for rejecting a null hypothesis of *β*_3 _= 0 for a given type I error rate (we set *α *= 0.05). The last term *e*_*i *_is the error part for individual *i *which follows a standard normal distribution *N(0,1)*. To assess the model performance, we specify different sample sizes and assign different values for *β*_3_. We set *β*_3 _to -0.65, -0.95 and -1.2 so that the interaction effect accounts for about 5, 10 and 15 percent of the total variance in the data.

In Table 1, we show the power estimates using 500 replicates for given type I error rate of *α *= 0.05. It can be seen that for an interaction effect that explains only 5 percent of the total variance (*β*_3 _= -0.65), a sample size of above 400 is needed in order to achieve reasonable power. For an effect responsible for 15 percent of the overall variance (*β*_3 _= -1.2), a sample size of 150 will give acceptable power (about 0.8). By setting *β*_3 _to zero, we further our simulation study to assess the type I error rate for a given nominal *α *= 0.05 using again 500 replicates. The estimated empirical type I error rate is shown in the right most column in Table 1. It can be seen that, although there is a slight fluctuation, the estimates of empirical type I error rate are centered at the nominal *α *of 0.05. Overall, results from our simulation study indicate that our retrospective model exhibits high power in capturing even relatively small interaction effect with reasonable sample sizes.

**Table 1:** Power and empirical type I error rate for given *α *= 0.05

Sample size	Power			Type I error
		
	*β*_3 _= -0.65	*β*_3 _= -0.95	*β*_3 _= -1.20	*β*_3 _= 0
150	0.348	0.642	0.780	0.052
200	0.540	0.668	0.894	0.048
250	0.604	0.852	0.898	0.054
300	0.664	0.884	0.960	0.046
400	0.760	0.948	1.000	0.050
600	0.876	1.000	1.000	0.052

## Results

The effect of catalase -262C/T promoter polymorphism on human aging phenotypes (cognitive and physical functioning) has been investigated by Christiansen et al. [15]. In this study, a modest protective effect of the T allele on cognitive and physical function was observed although a statistical significance was not reached. Here we apply our retrospective logistic regression model to the data to look for both main and interaction effects on individual's cognitive score (a continuous trait measuring fluency, for- and backward digit span and a modified 12-word learning test) by the genetic variant (T allele carrier = 1, non-carrier = 0) and age-group (equal or above age 65 = 1, below age 65 = 0) in males (N = 789). A combination of the two variants forms four nominal response categories among which non-carriers below age 65 serve as the reference group. In Table 2, we show the parameter estimates for the main and interaction effects by our logistic regression model. The model identified a highly significant effect of age-group that is negatively correlated with individual's cognitive function (RRR = 0.630, p-value = 0.001). Moreover, we found a modest main effect of the T allele (RRR = 0.948, p-value = 0.037) and a modest interaction effect between the T allele and age-group (RRR = 1.083, p-value = 0.033). It is interesting to see that, although the overall effect of allele T reduces carrier's cognitive score, the interaction effect indicates that the effect of the allele is age-dependent which means that the T allele conveys beneficial effect that improves carries' cognitive performances at old ages.

**Table 2:** Parameter estimates for main and interaction effects on cognitive score by the logistic regression model

	Slope	SE	p-value	Risk			logMLK*
					
				RRR	95% CI		
Continuous							
Age-group	-0.463	0.036	0.000	0.630	0.587	0.676	
Allele T	-0.054	0.026	0.037	0.948	0.901	0.997	
Interaction effect	0.079	0.037	0.033	1.083	1.006	1.164	
							-862.657
Dichotomous							
Age-group	-2.822	0.253	0.000	0.060	0.036	0.098	
Allele T	-0.411	0.202	0.041	0.663	0.446	0.984	
Interaction effect	0.870	0.333	0.009	2.386	1.241	4.588	
							-928.454

*MLK = maximum likelihood

By dichotomizing the cognitive score it is possible to apply the case-only model to assess the interaction effect of allele T and aging on cognitive functioning. To do that, we selected all individuals with cognitive score above 4 (about 24% of the top scores) and defined them as cases (186 individuals). The case-only model gave an odds ratio of 2.386 with a p-value of 0.008 indicating that allele T significantly enhances carrier's cognitive function at old ages. For comparison, we applied our retrospective logistic regression model to the dichotomized cognitive score. Parameter estimates in Table 2 also reveal the negative association with cognitive functioning by aging (RRR = 0.060, p = 0.000) and allele T (RRR = 0.663, p = 0.041). Meanwhile our model also reports a highly significant interaction effect even with exactly the same estimate of the risk parameter (RRR = 2.386, p = 0.009) as from the case-only model (OR = 2.386, p = 0.008) meaning that our retrospective logistic regression model yields valid estimate of the interaction effect. Consistent estimates on the interaction effect by the case-only and our models were also obtained when varying the cut-off for dichotomizing the cognitive score. This is understandable since the case-only model measures the deviation from the multiplication of main effects [9] which is exactly the definition of interaction effect in our model. However, since the maximum likelihood from the dichotomized trait is lower than the continuous trait (Table 2), the model using cognitive score as a continuous trait should be preferred.

## Discussion

The etiology of multifactorial human disease involves complex interactions between numerous environmental factors and alleles of many genes. Efficient statistical tools are demanded for identification of the genetic and environmental variants that affect the risk of disease development. Through example application, we have shown that our retrospective polytomous logistic regression model can capture both main and interaction effects and produce consistent results in estimating the interaction effect as compared with the popular case-only model. The distinct feature in our model is that the disease trait is treated as an independent variable so that our model is capable of accommodating both categorical and continuous traits. Different from the existing models [12,13], no assumption of normal distribution of the trait value is needed in our method. Furthermore, genotype or allele effects can be easily estimated by coding 1 and 0 to carriers and non-carriers to assess dominant or recessive effects.

Since our relative risk ratio is estimated from a retrospective model, it is necessary to study its connection with the relative risk parameter in a general prospective model. In additional file-1, we derive the relationship between the risk parameters in the retrospective model and that in the prospective model when studying a binary disease trait. It is shown that, when the disease is rare, the relative risks in a prospective model can be approximated by the relative risk ratios estimated from our model. This is important because, as long as the disease incidence is low in the population, our model estimates the risk parameters that can be interpreted in terms of trait penetrance as in a prospective model. As shown by equation (6), testing the null hypothesis of *b *= 0 is equivalent to testing *H*_*o *_: *RRR *= 1. This is also shown by the 95% confidence intervals for the estimated RRRs in Table 2. Since the slope parameters for the main and interaction effects are all statistically different from zero, none of the 95% confidence intervals of RRR covers the null risk of one.

It is necessary to point out that, as in any interaction model, it is critical that the interacting variants be independent. By independent we mean that the interacting variants are not correlated or in association. This is especially relevant in studying gene by gene interactions. It is important to make sure that the two loci under testing are not in LD if they reside on the same chromosome. In case of LD between the two genetic variants, a haplotype-based analysis is more appropriate [8]. Our experience showed that violation of independence can result in unreliable estimates on the risk parameters. Independence between interacting variables is also required by the case-only model to ensure reliable estimates [17]. For case-control studies, if the main interest is interaction effect, the case-only model should be preferred because it is more efficient than the traditional case-control model [18]. Note also that our model is limited to discrete exposure variables when applied to gene-environment interaction although it is no longer a problem for measuring gene by gene interactions because all genotypes are discrete.

Finally, although our model is proposed for genetic association studies (gene by gene or gene by environment), the same model can be applied to study the main and interaction effects of non-genetic variants. Perhaps the biggest advantage of our approach is that it can easily be implemented by using any programming statistical package to fit the multinomial logistic regression model. Considering all these advantages, we hope that our proposed method can be of use for epidemiologists who are interested in studying multifactorial or complex human diseases.

## Conclusion

Our proposed retrospective polytomous logistic regression model can be used as a convenient tool for assessing both main and interaction effects in genetic association studies of human complex diseases involving both genetic and non-genetic factors.

## Authors' contributions

QT designed the study, analyzed the data and drafted the manuscript. LC and SL contributed to the study design and provided data. CBA and JHZ contributed to the formulation and design of the study. TAK and KC directed the study. All authors approved the final manuscript.

## Supplementary Material

Additional file 1 The relationship between risk estimates in the retrospective and the prospective models. In this additional file, we derive the relationship between risk estimates in the retrospective and the prospective models under the assumption of low incidence for a binary disease trait. Click here for file
