# Supplementary material for: Retrospective analysis of main and interaction effects in genetic association studies of human complex traits
Source: BMC Genet. 2007 Oct 16;8:70. doi: 10.1186/1471-2156-8-70 (PMC2099440; doi:10.1186/1471-2156-8-70)
Supplement: Additional file 1 — The relationship between risk estimates in the retrospective and the prospective models. In this additional file, we derive the relationship between risk estimates in the retrospective and the prospective models under the assumption of low incidence for a binary disease trait. [file 1471-2156-8-70-S1.doc]

**The relationship between risk estimates in the retrospective and the prospective models**

Suppose we are interested in studying the main and interaction effects for a genetic variant (G) and an environmental factor (E) in a binary disease trait with $x=1$for the affected and$x=0$for the unaffected. Let $N$ be the size of the population from which the samples were taken and P be the disease prevalence in the population. We present the entire population in the following table in which the total population is divided according to their disease status conditional on the exposure and genotype. In the table, $p(G,E|x)$ is the frequency of the subpopulation having genotype G and exposure E with disease status$x$. Applying (8) and (9) to the table, we calculate the overall RRR for carriers who are exposed as

$RRR=\frac{p(G+,E+|1)}{p(G-,E-|1)}/\frac{p(G+,E+|0)}{p(G-,E-|0)}=\frac{p(G+,E+|1)p(G-,E-|0)}{p(G-,E-|1)p(G+,E+|0)}$. (1’)

Now, for the entire population, the rate of disease among the exposed carriers is

$$p(D|G+,E+)=\frac{p(G+,E+|1)PN}{\left[p(G+,E+|1)P+p(G+,E+|0)(1-P)\right]N}=\frac{p(G+,E+|1)P}{p(G+,E+|1)P+p(G+,E+|0)(1-P)}$$

(2’)

Similarly, the rate of disease among the unexposed non-carriers is

$p(D|G-,E-)=\frac{p(G-,E-|1)P}{p(G-,E-|1)P+p(G-,E-|0)(1-P)}$. (3’)

From (2’) and (3’), we obtain the overall relative risk for the exposed carriers in a standard prospective model,

$$\begin{matrix} RR=\frac{p(D|G+,E+)}{p(D|G-,E-)}=\frac{p(G+,E+|1)P}{p(G+,E+|1)P+p(G+,E+|0)(1-P)}/\frac{p(G-,E-|1)P}{p(G-,E-|1)+p(G-,E-|0)(1-p)} \\ \\ \begin{matrix} \begin{matrix} \begin{matrix} \end{matrix} \end{matrix} \end{matrix}\begin{matrix} & = \end{matrix}\frac{p(G+,E+|1)}{p(G+,E+|1)P+p(G+,E+|0)(1-P)}\times\frac{p(G-,E-|1)P+p(G-,E-|0)(1-P)}{p(G-,E-|1)} \end{matrix}$$

(4’)

When P is small, we have in (4’), $p(G+,E+|1)P+p(G+,E+|0)(1-P)\cong p(G+,E+|0)$ and$p(G-,E-|1)P+p(G-,E-|0)(1-P)\cong p(G-,E-|0)$. With this approximation, we have

$RR\approx\frac{p(G+,E+|1)p(G-,E-|0)}{p(G+,E+|0)p(G-E-|1)}=RRR$. (5’)

By applying (5) and (6), we can derive the relative risk ratio for the main genetic effect as

$RRR_{G}=\frac{p(G+,E-|1)}{p(G-,E-|1)}/\frac{p(G+,E-|0)}{p(G-,E-|0)}=\frac{p(G+,E-|1)p(G-,E-|0)}{p(G-,E-|1)p(G+,E-|0)}$. (6’)

For the entire population, the rate of disease among the unexposed carriers is

$$p(D|G+,E-)=\frac{p(G+,E-|1)PN}{\left[p(G+,E-|1)P+p(G+,E-|0)(1-P)\right]N}=\frac{p(G+,E-|1)P}{p(G+,E-|1)P+p(G+,E-|0)(1-P)}$$

(7’)

From (7’) and (3’), we obtain the relative risk for the carriers in a prospective model,

$$\begin{matrix} RR_{G}=\frac{p(D|G+,E-)}{p(D|G-,E-)}=\frac{p(G+,E-|1)P}{p(G+,E-|1)P+p(G+,E-|0)(1-P)}/\frac{p(G-,E-|1)P}{p(G-,E-|1)+p(G-,E-|0)(1-p)} \\ \\ \begin{matrix} \begin{matrix} \begin{matrix} \end{matrix} \end{matrix} \end{matrix}\begin{matrix} & = \end{matrix}\frac{p(G+,E-|1)}{p(G+,E-|1)P+p(G+,E-|0)(1-P)}\times\frac{p(G-,E-|1)P+p(G-,E-|0)(1-P)}{p(G-,E-|1)} \end{matrix}$$

(8’)

With small P, we have$p(G+,E-|1)P+p(G+,E-|0)(1-P)\cong p(G+,E-|0)$ and$p(G-,E-|1)P+p(G-,E-|0)(1-P)\cong p(G-,E-|0)$. Now (8’) can be reduced to

$RR_{G}\approx\frac{p(G+,E-|1)p(G-,E-|0)}{p(G+,E-|0)p(G-E-|1)}=RRR_{G}$. (9’)

Likewise, the same approximation applies to the main effect of environment, i.e. $RR_{E}\approx RRR_{E}$. (10’)

Since the interaction effect, by definition, measures the departure from the multiplicative effects from the main factors both in the prospective and the retrospective models, we have $RR_{GxE}=\frac{RR}{RR_{G}RR_{E}}$ in the prospective model and$RRR_{GxE}=\frac{RRR}{RRR_{G}RRR_{E}}$ in the retrospective model. Given the relationships in (5’), (9’) and (10’), we obtain

$RR_{GxE}\approx RRR_{GxE}$. (11’)

With these connections, the relative risk parameters in the prospective model can be approximated by the RRRs in our retrospective model under the condition of low disease incidence.

|  |  | Disease status | |  |
| --- | --- | --- | --- | --- |
|  |  | Case ($x=1$) | Control ($x=0$) | Total |
| Exposure  E+ | Carrier  G+ | $p(G+,E+\vert1)PN$ | $p(G+,E+\vert0)(1-P)N$ | $\left[p(G+,E+\vert1)P+p(G+,E+\vert0)(1-P)\right]N$ |
|  | Non-carrier G- | $p(G-,E+\vert1)PN$ | $p(G-,E+\vert0)(1-P)N$ | $\left[p(G-,E+\vert1)P+p(G-,E+\vert0)(1-P)\right]N$ |
| Unexposed  E- | Carrier  G+ | $p(G+,E-\vert1)PN$ | $p(G+,E-\vert0)(1-P)N$ | $\left[p(G+,E-\vert1)P+p(G+,E-\vert0)(1-P)\right]N$ |
|  | Non-carrier G- | $p(G-,E-\vert1)PN$ | $p(G-,E-\vert0)(1-P)N$ | $\left[p(G-,E-\vert1)P+p(G-,E-\vert0)(1-P)\right]N$ |
| Total | | $PN$ | $(1-P)N$ | $N$ |
